# The Worst War of the World

**Published:** 1917-08-18

**Authors:** 


### THE WORST WAR OF THE WORLD.

### III. THE UNITED STATES TROOPS VISIT LONDON.

In an article we published in The Hospital of August 8, 1914, entitled "War and Insanity," speaking of Germany's hurried declaration of war and the evidence laid before the House of Commons by the Government on August 3 and 4 in that year, we pointed out that "this evidence demonstrated once more that madness may precede destruction when the gods take action. Is it conceivable otherwise that any man of intelligence and authority could persuade himself that he had behind him sufficient money, weapons of destruction, and fists, to compel the whole world and the people it contains, to fall down and worship him at his will and pleasure? Yet this belief seems to dominate some of the contestants, or they could not have brought themselves publicly to shed all the high standards of conduct, religious sentiment, and principles which they have heretofore publicly claimed to possess. The patient, in his haste to capture the world, declared war upon his chief neighbour directly he consented to negotiate for peace. This done, every possible device and insult was resorted to to bring first one great Power and then another into the strife. When these attempts failed he declared war against them all. But our patient could not be content! He had got a plan of what he wished to do, and how it could be made successful. He accordingly broke the honourable engagements into which he had entered, tore up the documents, and proceeded to bring himself into a state of war with small and great. The civilised world, first shocked and horrified, is now uniting, and has placed the patient under strict observation. There can be no doubt that any war, thus brought about, is not only a mean war, but a war originating in madness. The world must realise at the outset, by the facts now known to all the peoples, that the real authors of the present European war, their objects, actions, and ambitions, afford evidence to justify a properly constituted Board of Lunacy in placing them in strict confinement for their own safety and the safety of other people."

"The present war, then, originated in moral degeneracy and the absence of sound judgment. Its authors persuaded themselves that the money they had accumulated, the armaments and munitions of war they had created, and the control of the enormous armies they had drilled to the use of these implements of destruction, would prove irresistible. A lunatic always believes that he is irresistible, and not frequently claims to be the Deity Himself. It will be plain to all thoughtful minds that the nations of the earth should combine and quickly stamp out this war by placing the authors of it in safe confinement out of mischief. They can never be permitted to control, to rule, and to enslave the world."

This forecast, written immediately after the declaration of war by Germany, it will be universally agreed, has been remarkably fulfilled. Ambassador Gerard's book, now being published in the *Daily Telegraph*, "My Four Years in Germany," by James W. Gerard, late Ambassador in Berlin, as it proceeds day by day confirms and strengthens its accuracy.

August 15, 1917, will be memorable in the history of the world. Also a great day of re-uniting and brotherhood for the people of the United States and Great Britain. That day the first contingent of American troops paid a visit to London to enable the British public to give them welcome. They numbered some 4,000 men, and marched through some of the chief thoroughfares of the West End, leaving Wellington Barracks at 11.20 a.m., and proceeding by the Horse Guards' Parade, through Whitehall into Trafalgar Square; on through Cockspur Street and Pall Mall to St. James's Street, then along Piccadilly, through Grosvenor Place to Grosvenor Gardens, where they passed the United States Ambassador at the Embassy, and so on to Buckingham Palace, at the entrance to which stood His Majesty King George V., with an escort of British troops in full kit, and, passing His Majesty, marched into the Green Park, where they were entertained in an informal way as the guests of our Guards' regiments, who took them under their kindly wing and gave them a hearty welcome from the British Army. After a short halt the United States troops resumed their march through the Mall, across the Horse Guards Parade, thence through Whitehall, over Westminster Bridge, down Westminster Bridge Road, to Waterloo Station. It had been hoped that a larger extent of hospitality could have been offered to both officers and men, but, after consultation with the American Embassy, it was found, for military reasons, to be impossible. The arrangements made met with the full approval of the American Embassy. The American flag was flown from the Houses of Parliament, the Clock Tower, the Government and other buildings along the route.

The Royal Standard, with the assistance of the wind, was magnificently displayed from the flagstaff immediately behind the position where His Majesty with Queen Alexandra and Princess Mary stood at the entrance to Buckingham Palace, as the American troops marched past at the salute. Our cousins, the Americans, though democrats, share with ourselves our feeling of respect and loyalty for King George, and will remember with pride the fact that the Head of the Great Nation, with whom the United States is now allied in a holy cause, has stood at the door of his own house to give the American troops a kingly salute. The Memorial to Queen Victoria was crowded with people, as were the approaches to Buckingham Palace. The cheering was continuous, increasing to enthusiasm as each regiment marched past led by the bands of the Brigade of Guards and the pipes.

Throughout, the scene en route was inspiriting, attractive and convincing. Inspiriting because of the large crowds of welcome, who were impressed with the interest and importance of the occasion, and, though enthusiastic, became so absorbed in

☛ Articles I. and II. appeared July 28 and August 11.

**THE WORST WAR IN THE WORLD** (*continued*). the magnificent and continuous display of some of the best equipped and most stalwart fighting men in the world, that though the hand-clapping and handkerchief-waving was continuous, the outburst of cheering was intermittent in the streets, marked by the taking off of hats to the Colours, and by special cheers as the first troops of each regiment reached one of the groups into which the crowds were divided.

It was a splendid day. There was a grim, forcible determination, under a very modest demeanour, in the faces of the United States troops, as they marched from the Horse Guards Parade to the Green Park. On leaving the Green Park, on their return to Waterloo, the Americans had evidently been warmed by the welcome of their British comrades in arms, and pleased by their reception in the streets, for the faces of many of them showed that they were happy, inspirited, and full of rejoicing, as their cousins, the people of London, were too, at this uniting of the Anglo-Saxon race in a holy cause, fraught with momentous issues for the civilised world. It matters not what the All-Highest and the Germans' responsible for the Great War may think of this union of hearts and peoples. But as the lunatic is blessed occasionally with sane moments of clear apprehension, we may conclude that the authors of the war, as one result of the proceedings in London on the 15th August, 1917, the fourth year of the war, may begin to realise, in some small degree at any rate, the madness which led them to set out to conquer the world and enslave it, so recently as 4th August, 1914. There is a peculiar fitness in this connection, that the *Daily Telegraph* of the 15th instant published, in the twelfth chapter of Mr. Gerard's book, an account of an interview with the Emperor William at the Kaiser's Château on May 1, 1916, which took place during the Ambassador's stay at General Headquarters. At this interview the Emperor referred to the efforts to starve Germany and keep out milk from that nation, and declared that, before he would allow his family and grandchildren to starve, he would blow up Windsor Castle and the whole Royal Family in England.

